# The prevalence of norovirus, astrovirus and adenovirus infections among hospitalised children with acute gastroenteritis in Porto Velho, state of Rondônia, western Brazilian Amazon

**DOI:** 10.1590/0074-02760140381

**Published:** 2015-04

**Authors:** Maria Sandra Costa Amaral, Grecy Kelli Estevam, Marilene Penatti, Roger Lafontaine, Ian Carlos Gomes Lima, Paula Katharine Pontes Spada, Yvone Benchimol Gabbay, Najla Benevides Matos

**Affiliations:** 1 Insituto Oswaldo Cruz-Fiocruz, Porto Velho, RO, Brasil; 2 Hospital Infantil Cosme e Damião, Secretaria de Estado da Saúde, Porto Velho, RO, Brasil; 3 Centro de Pesquisa em Medicina Tropical, Porto Velho, RO, Brasil; 4 Instituto Evandro Chagas, Belém, PA, Brasil

**Keywords:** acute gastroenteritis, children, gastroenteric viruses

## Abstract

Although viruses are well-established causes of acute gastroenteritis, few data on the circulation of these pathogens in Porto Velho, state of Rondônia, Brazil, are available. Thus, faecal samples from hospitalised diarrhoeic children, under six years of age, were collected and tested for the presence of norovirus (NoV), adenovirus (AdV) and astrovirus (AstV) from February 2010-February 2012. Specimens were screened by reverse-transcription polymerase chain reaction and viruses were found in 10.7% (63/591) of the cases. NoV, AdV and AstV were detected in 7.8%, 2% and 0.8% of the samples, respectively. NoV infection was observed at all ages and was most prevalent in zero-18-month-old children (84.7%; p = 0.002). A higher incidence of NoV was detected from February-April 2010, when it was found in 52.2% of the cases. Co-infections involving these viruses, rotavirus and enteropathogenic bacteria were detected in 44.4% (28/63) of the children with viral diarrhoea. Nosocomial infections were demonstrated in 28.6% (18/63) of the cases in which viruses were detected. The present paper reports, for the first time, the circulation of NoV and AstV among the paediatric population of Porto Velho and it contributes to our understanding of the roles of these pathogens in gastrointestinal infections.

Viruses are considered to be the major cause of acute diarrhoea in young children. Four categories of viruses are considered to be important agents of viral diarrhoea: group A rotavirus (RVA) (Reoviridae family), norovirus (NoV) (Caliciviridae family), adenovirus (AdV) (Adenoviridae family), mainly serotype 40/41 (species F) and astrovirus (AstV) (Astroviridae family). The medical importance of RVA is well established and, for this reason, since March of 2006, the Rotarix^(r)^ vaccine has been included in the Brazilian National Immunisation Program (NIP). There has been a subsequent reduction in hospital admissions due to gastroenteritis in most regions of Brazil, most likely due to the efficacy of this vaccine (Borges et al. 2011, do Carmo et al. 2011 , Sandra et al. 2013).

Studies have demonstrated that NoV is the most important aetiological agent of acute non-bacterial gastroenteritis outbreaks, which are transmitted mainly by contaminated food and water; NoV affects adults and children worldwide (Patel et al. 2008). NoV infections are of critical importance in developing countries, where NoV is responsible for up to 1.1 million hospitalisations and causes approximately 218,000 deaths per year (Patel et al. 2008).

Human AstV (HAstV) is an important pathogen in the aetiology of diarrhoea in communities and it can cause serious infections that require medical care and hospitalisation (Gabbay et al. 2005, Jeong et al. 2012).

Currently, enteric human AdV (HAdV) are considered to be the third leading cause of non-bacterial diarrhoea among children, in addition to being one of the primary agents responsible for paediatric intussusception caused by viral agents (Barrella et al. 2009, Minney-Smith et al. 2014).

The presence of HAdV in water used for drinking and recreation has been linked to persistent infections and outbreaks (Barrella et al. 2009). This virus, together with bacteria, has been singled out as a future indicator of microbiological water quality. Several HAdV outbreaks have been described in primary schools, hospitals, military barracks and recreational water facilities (Jiang 2006 ).

In a previous study in Porto Velho, state of Rondônia (RO), in the western Brazilian Amazon, data on the aetiology of acute gastroenteritis was gathered in hospitalised children under five years of age from March 2000-March 2002; RVA was detected with a frequency of 23.6% and HAdV was detected with a frequency of 6.4% (Magalhães et al. 2007). RVA-positive samples were also genotyped (Costa et al. 2012). Given the need to establish profiles of aetiological agents of acute gastroenteritis, the present study aimed to detect enteric viruses in stool samples from children admitted to a paediatric hospital in Porto Velho.

## SUBJECTS, MATERIALS AND METHODS

*Location of study and patients* - A total of 591 samples collected from February 2010-February 2012 were analysed. Faecal samples were obtained from children up to six years of age who were hospitalised with acute gastroenteritis at the Cosme and Damião Children's Hospital, a public tertiary care institution in Porto Velho. In addition, children hospitalised with other symptoms, such as respiratory symptoms, who developed gastroenteritis during their stay in the hospital were included in this study as nosocomial cases.

Acute gastroenteritis cases were defined as having liquid or semi-liquid stools, with three or more evacuations in a 24-h period. Sample collection was performed three times per week, always on Mondays, Wednesdays and Fridays, for two years without interruption. Only one sample was collected from each child. The faecal samples were collected using a sterile universal collector. Samples were registered, labelled and stored at -80ºC at the Microbiology Laboratory of the Tropical Medicine Research Centre. The study was approved by the Ethical Committee of Rondônia Tropical Medicine Research Centre (protocol 0113/2010).

*Bacteriology* - All specimens were processed using routine microbiological and biochemical tests obtained from bioMérieux, France (API 20E) to identify *Escherichia coli*, *Salmonella* spp and *Shigella* spp strains that were selected from Salmonella-Shigella agar (HiMedia), xylose lysine deoxycholate agar (HiMedia) and Brilliant Green agar (HiMedia).

*Analysis of E. coli virulence factors by multiplex polymerase chain reaction (PCR)* - To determine whether diarrhoeagenic *E. coli *was present in the selected subcultures, multiplex PCR tests were conducted as described previously (Muller et al. 2007 ).

*Virology* - The same samples were also assayed for the presence of rotaviruses (RVs) using a commercial enzyme-linked immunosorbent assay (ELISA) (Rotaclone^(r))^ following the manufacturer's instructions. Molecular techniques were used to determine RVA electrophoretic profiles and RVA-positive samples were genotyped by reverse-transcription PCR (RT-PCR) followed by multiplex nested-PCR (Sandra et al. 2013).

*Viral DNA and RNA extraction* - A commercial kit (QIAamp DNA stool kit, Qiagen, Germany) was used to extract viral DNA from stool specimens and the TRIzol method (Gibco-BRL; Life Technologies(tm), USA) was used for RNA extraction according to the manufacturer's instruction.

*Molecular detection* - The specimens were screened by RT-PCR using the Mon 340/348 (Belliot et al. 1997), Mon 269/Mon270 (Noel et al. 1995) and precap1/82b (Yan et al. 2003 ) primers for HAstV and the p289/p290 primers for human calicivirus (Jiang et al. 1999). NoV was initially detected by PCR with the primers Mon 431/432 and 433/434, by nested PCR using the JV12Y/ JV13I primers in the first step and the JV13I/GI or JV12y/NoroII-R primers for NoV genogroups I and II, respectively, in the second step (Fankhauser et al. 2002, Vennema et al. 2002).

Nested PCR was also performed for HAdV using the HEX1DEG and HEX2DEG primers (1st step) followed by the NEHEX3DEG and NEHEX4DEG primers (2nd step) (Allard et al. 2001).

All PCR products were analysed on 1.5% agarose gels in the presence of SYBR^(r)^ Safe and the gels were visualised under ultraviolet light.

*Statistical analysis* - Statistical analyses of the data were performed using classical methods in GraphPad Prism 5.0^(c)^. The data were subjected to Fisher's exact test and odd ratios were calculated, together with 95% confidence intervals. A value of p < 0.05 in bi-tailed tests was considered statistically significant in all analyses.

## RESULTS

From February 2010-February 2012, viral agents were detected in 10.7% (63/591) of the samples from children under six years of age with acute gastroenteritis symptoms. NoV, HAdV and HAstV were detected in 7.8% (46/591), 2% (12/591) and 0.8% (5/591) of the samples, respectively. A total of 17.4% (103/591) of samples were determined to be positive for RVA by ELISA. These samples were also subjected to polyacrylamide gel electrophoresis analysis to confirm the results, as well as to observe the electrophoretic profile of the viral genome (Sandra et al. 2013). Of the 103 RVA-positive samples, 52 samples were characterised by both G and P typing. The most prevalent genotype G9P[8] was detected in 94.2% (49/52) of the samples, followed by G2P[4] at 3.9% (2/52) and G1P[8] at 1.9% (1/52) (Sandra et al. 2013).

RVA infection was mainly observed in zero-18-month-old (58%; p = 0,007) and > 24-month-old children (25.2%; p = 0.004). NoV infections were observed at all ages and were most prevalent in zero-18-month-old children (p = 0.002). HAdV was detected at equal rates in all age groups, while HAstV was detected only in seven-18-month-old children (Table I).

**TABLE I.** Distribution by age group of the positive cases of norovirus (NoV), astrovirus (AstV), adenovirus (AdV) and rotavirus (RVA) in children with acute gastroenteritis in Porto Velho, state of Rondônia, Brazil, February 2010-February 2012

Age months (n)	NoV				AstV				AdV				RVA^*a*^		
	n (%)	p^*b*^	OR		n (%)	p	OR		n (%)	p	OR		n (%)	p	OR
0-6 (134)	9 (19.6)	0.143	0.817		0 (0)	0.119	0.306		4 (23.5)	0.200	1.051		16 (15.5)	0.014	0.577
7-12 (161)	18 (39.1)	0.017	1.807		2 (40)	0.123	1.790		3 (17.6)	0.116	0.564		25 (24.3)	0.109	0.830
13-18 (103)	12 (26.1)	0.022	1.761		3 (60)	0.008	7.290		3 (17.6)	0.200	1.016		19 (18.4)	0.155	1.088
19-24 (91)	4 (8.7)	0.057	0.501		0 (0)	0.200	0.492		4 (23.5)	0.063	1.722		17 (16.5)	0.153	1.106
> 24 (102)	3 (6.5)	0.009	0.314		0 (0)	0.119	0.430		3 (17.6)	0.200	1.028		26 (25.2)	0.004	1.830
Total (591)	46 (100)	-	-		5 (100)	-	-		17 (100)	-	-		103 (100)	-	-

a : Sandra et al. (2014);

b : Fisher's exact test with Bonferroni correction (α = 0.01). When we analysed the grouped age group, it was observed a statistically significant relation in NoV of 0-18 month children [p = 0.002; odds ratio (OR) = 2.887, 95% confidence interval 1.266-6.580] by using Fisher's exact test with Bonferroni correction. In all RVA comparisons (0-12; 0-18; 0-24) statistically significant values were observed, without considerable OR values.

Diarrhoeagenic *E. coli*, *Salmonella* spp and *Shigella* spp were the enteropathogenic bacteria found most often in acute gastroenteritis, at rates of 22.8% (135/591), 7% (42/591) and 2% (13/591), respectively.

Co-infections involving these viruses, RVA and enteropathogenic bacteria were detected in 46% (29/63) of the children with viral diarrhoea (Table II). The following co-infections were observed most frequently: NoV and enteroaggregative *E. coli* (EAEC) in 24.1% (7/29) of the cases, NoV and RVA in 20.7% (6/29) of the cases and NoV and atypical enteropathogenic *E. coli* in 10.3% (3/29) of the cases. Co-infections with HAdV and RVA were observed in 6.9% (2/29) of the cases and co-infections by HAdV and enteropathogenic bacteria were observed in 20.7% (6/29) of the cases. Two cases (6.9%) of triple infection were observed; HAdV, EAEC and *Salmonella *spp or RVA were detected in these cases.

**TABLE II.** Clinical features observed in each virus with combinations of viruses and in mixed infections

		Symptom		
	n	Bloody diarrhoea n (%)	Vomiting n (%)	Fever n (%)
NoV	26	3 (11.5)	21 (80.7)	17 (65.3)
AstV	2	0 (0)	2 (100)	1 (50)
AdV	4	1 (25)	4 (100)	4 (100)
NoV + RVA	6	1 (16.6)	5 (83.3)	5 (83.3)
NoV + AstV	2	0 (0)	2 (100)	1 (50)
NoV + EAEC	7	2 (28.5)	6 (85.7)	5 (71.4)
NoV + tEPEC	1	0 (0)	1 (100)	0 (0)
NoV + aEPEC	3	0 (0)	3 (100)	1 (33.3)
NoV + EHEC	1	0 (0)	0 (0)	1 (100)
AstV + EAEC	1	0 (0)	0 (0)	1 (100)
AdV + RVA	2	0 (0)	2 (100)	2 (100)
AdV + RVA + EAEC	1	1 (100)	1 (100)	1 (100)
AdV + tEPEC	1	0 (0)	1 (100)	1 (100)
AdV + aEPEC	1	0 (0)	1 (100)	1 (100)
AdV +* Salmonella *spp	2	0 (0)	2 (100)	2 (100)
AdV + EAEC + *Salmonella *spp	1	0 (0)	0 (0)	0 (0)

there was no statistically significant difference between single infections and co-infections in each studied virus. AdV: adenovirus; aEPEC: atypical enteropathogenic Escherichia coli; AstV: astrovirus; EAEC: enteroaggregative E. coli; EHEC: enterohaemorrhagic E. coli; NoV: norovirus; RVA: rotavirus; tEPEC: typical enteropathogenic E. coli.

The major clinical symptoms related to viral gastroenteritis in the current study are shown in Table II. All children had typical symptoms associated with viral infection, including diarrhoea, vomiting and fever. Bloody diarrhoea was observed in 12.6% (8/63) of the cases, 11.5% (3/26) of which were positive for NoV and 25% (1/4) had HAdV infections. Bloody diarrhoea was also observed in co-infections with dual viral infection or viral and bacterial infection, especially when enteropathogenic *E. coli* was the implicated agent, which was observed in 13.8% (4/29) of the cases. Viral infections exhibited seasonality, as they were mainly detected from February-June, which is the rainy season. Higher incidences of NoV were detected from February-April 2010, during which it was found in 52.2% (24/46) of the positive cases; NoV was detected at lower rates in the subsequent months. The incidence of RVA was higher from February-September 2010, during which it was found in 75.7% (78/103) of samples; however, the number of cases positive for RVA decreased in the second year (Figure).

**Figure:**
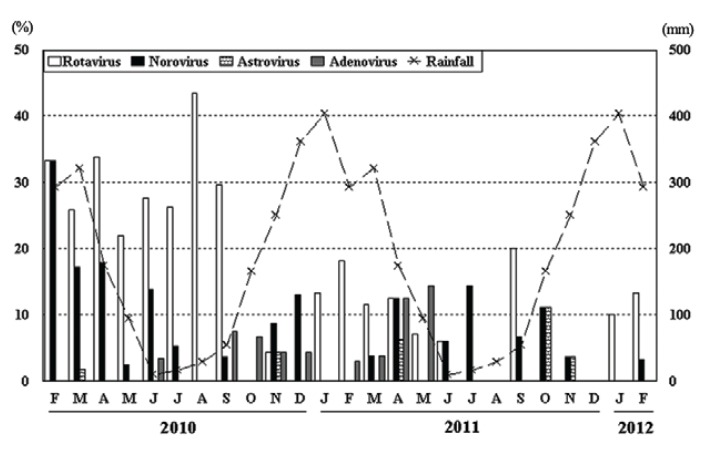
Monthly distribution of norovirus, astrovirus, adenovirus and rotavirus in 591 faecal samples collected from diarrheic children, Porto Velho, state of Rondônia, Brazil, February 2010-February 2012.

In the current study, 28.6% (18/63) of the viral gastroenteritis cases that were investigated were nosocomial. A total of 26.1% (12/46) of these cases were positive for NoV, 16.7% (2/12) were positive for HAdV and 80% (4/5) were positive for HAstV (Table III). No specific clustering of nosocomial infections was identified.

**TABLE III.** Cases of nosocomial infections detected during the study period

Virus	Positive/total n/n (%)	Clinical characteristics^*a*^
Norovirus	12 /46 (26.1)	Respiratory infection(9) Fall (1) Infection in the blood (1) Neurological problem (1)
Adenovirus	2/12 (23.5)	Respiratory infection (2)
Astrovirus	4 /5 (80)	Respiratory infection (4)

a : causes of hospitalisation before acute gastroenteritis.

## DISCUSSION

In the present study, NoV was determined to be responsible for 7.8% (46/591) of the gastrointestinal infections in hospitalised children using two different sets of primers, one specific for the RNA polymerase (region B, ORF1) and the other specific for the capsid (region D, ORF2). A previous study conducted in Europe also showed that NoV is an important cause of acute gastroenteritis in cases that required hospitalisation (Junquera et al. 2009, Levidiotou et al. 2009, Tran et al. 2010 ). Studies carried out in the states of Espírito Santo and Rio de Janeiro (RJ), Brazil, demonstrated higher NoV infection rates than those found in the current study (39.7% and 30.3%, respectively) (Ribeiro et al. 2008, Ferreira et al. 2012 c). Investigations conducted in a day-care centre in RJ over a 15-year period demonstrated that NoV was more prevalent than RVA and HAstV (Ferreira et al. 2012b). In a study of paediatric diarrhoeic children in Nicaragua, a prevalence rate of 24% (79/330) was found for NoV infection (Bucardo et al. 2014 ). After the RVA vaccine was implemented in Nicaragua, NoV became the main viral cause of acute gastroenteritis among children younger than five years of age (Bucardo et al. 2014). Similar results have been reported in the United States of America (Koo et al. 2012, Payne et al. 2013).

Considering that this is the first study conducted in Porto Velho to include NoV and that a positivity rate of 7.8% was observed for the entire research period overall, our results demonstrate the importance of such viruses as a cause of paediatric gastroenteritis. However, further studies of this virus are necessary, especially after the introduction of the Rotarix^(r)^ vaccine, which was correlated with an increase in the incidence of other viruses in many countries. Further studies are also necessary to investigate the use of other, more sensitive methods for detecting these agents (Wikswo et al. 2013, Rooney et al. 2014).

HAdV has been detected in gastroenteritis studies in developing and developed countries with a prevalence ranging from 2-35% (Bon et al. 1999, Pereira Filho et al. 2007, Andreasi et al. 2008, Verma et al. 2009, Muller 2010). In the present study, the percentage of samples positive for HAdV (2%) was lower than the 6.4% measured previously in Porto Velho from 2000-2002 (Magalhães et al. 2007).

The present report is the first to describe the presence of HAstV in Porto Velho. However, the 0.8% prevalence detected in the current study was lower than that observed previously in other studies conducted in Brazil (Andreasi et al. 2008, Ferreira et al. 2012a, Jeong et al. 2012), such as in São Luis, state of Maranhão, where HAstV was detected in 11% of the children with diarrhoea symptoms and in 5% of the children without symptoms (Gabbay et al. 2005). The prevalence observed in the current study was also lower than that (12.4%) reported previously in children in Argentina (Giordano et al. 2004, Maham et al. 2013). However, similar results (1.6%) were obtained in a study developed in Iran of 2,490 faecal samples collected from children with gastroenteritis. The prevalence of these viruses are most likely related to the geographical features and socioeconomic conditions of each country (Giordano et al. 2004, Gabbay et al. 2005, Andreasi et al. 2008, Malasao et al. 2012, Pativada et al. 2012).

Due to the morbidity and mortality rates associated with diarrhoea caused by gastrointestinal viruses and by RVA in particular, urgent measures were enacted in Brazil, such as the introduction of the Rotarix^(r)^ vaccine as part of the Brazilian NIP. The Rotarix^(r)^ vaccine has helped to reduce the number of hospitalisations caused by this agent, as has been demonstrated in recent reports (Safadi et al. 2010, do Carmo et al. 2011, Lanzieri et al. 2011, Sandra et al. 2013). A study conducted in five Brazilian Regions using the National Rotavirus Acute Diarrhoea Surveillance System showed the effectiveness of the RVA vaccine against acute diarrhoea in hospitalised children. This study documented a reduction in the rate of child hospitalisation and mortality due to acute diarrhoea in Brazil (Ichihara et al. 2014). However, more intensive monitoring is necessary in Porto Velho to better assess the real incidence of this and other viruses, such as NoV, that have exhibited increased incidences simultaneously with the decline in the incidence of RVA (Bucardo et al. 2008).

Many studies have demonstrated that NoV infection mainly occurs in children younger than two years old (Ribeiro et al. 2008, Aragão et al. 2010, Siqueira et al. 2013). In the present study, the highest NoV infection rates occurred in zero-18-month-old children (p = 0.022). However, in the current study, no statistically significant difference in the prevalence of the other viruses between age groups was observed.

In the present study, co-infections with NoV, HAdV and HAstV with RVA and enteropathogenic bacteria were observed in 46% of the cases investigated, most of which involved either NoV and EAEC or NoV and RVA. In agreement with these data, in a study conducted in León, Nicaragua, NoV was detected in 57% (37/65) of the cases examined and co-infections mainly involved NoV and enteropathogenic *E. coli *(Bucardo et al. 2008). Other studies identified co-infections with NoV and RVA at frequencies ranging from 2.1-50% (Oh et al. 2003 , Medici et al. 2004, Tran et al. 2010, Ferreira et al. 2012a). In the state of São Paulo, NoV co-infection was established with RVA, HAdV and HAstV at frequencies ranging from 1.8-11.1% (Castilho et al. 2006, Ferreira et al. 2012a). In Spain, 14% (7/51) of children hospitalised with acute gastroenteritis had co-infections; in that study, RVA and AstV were the most frequently observed agents (García-Magán et al. 2013). Currently, limited information is available about co-infection in RO and this phenomenon should be further investigated in this state and in Brazil.

In the current study, the major clinical symptoms associated with viral gastroenteritis infection, besides diarrhoea, were vomiting and fever. This observation is in agreement with findings obtained in other studies conducted in different countries, including Iran (Hamkar et al. 2010), France (Bon et al. 1999) and Brazil (Gabbay et al. 2005). As reported elsewhere, bloody diarrhoea was observed less frequently in children with viral gastroenteritis and there is a strong suspicion that such episodes may be associated with pathogenic bacteria. We did not detect significant differences in these symptoms among co-infection cases, but the number of cases in each group was too small to draw any conclusions.

More NoV infections were observed in 2010 (11% or 36/326) than in 2011 (3.8% or 10/265). In addition, most of the 2010 cases were detected between February-April (33.3%, 17.2% and 17.9%, respectively, corresponding to 52.2% of the detected cases). Few samples were genotyped; most of these samples were classified as NoV variant GII.4 New Orleans, 2009 (data not shown). It is possible that these data show the introduction of a new variant and such a new variant could have caused the observed increase in the number of NoV cases.

In a study conducted in Belém, state of Pará, in the North Region of Brazil (Siqueira et al. 2013), a higher frequency of NoV was also documented in the first year of follow-up.

The greatest number of RVA cases was observed in 2010, but the frequency of detected cases decreased in the following year. These data still suggest that there was a decrease in the prevalence of RVA after the introduction of the RVA vaccine by the Brazilian NIP. Nosocomial infections were identified in 18 (28.6%) of the 63 positive cases and were associated with NoV (n = 12), HAstV (n = 4) and HAdV (n = 2). Previous reports have demonstrated that NoV is a common cause of nosocomial diarrhoea in paediatric populations admitted to hospitals (Tran et al. 2010, Ferreira et al. 2012a). Additionally, a study performed in developed countries documented nosocomial infections by HAstV (Shastri et al. 1998). To better investigate nosocomial infections with HAdV, we sequenced and confirmed one isolate as HAdV type 41 (data not shown). All of these viruses survive for extended periods under adverse environmental conditions and they are directly associated with diarrhoeic infections (Bruijning-Verhagen et al. 2012).

In conclusion, the data presented in the current study may contribute to a better understanding of the role that gastrointestinal viruses play in childhood diarrhoeal illnesses in Porto Velho and may aid in strategic planning for controlling the disease in such regions.
